# Pharmacists and COVID-19

**DOI:** 10.1186/s40545-020-00241-3

**Published:** 2020-06-19

**Authors:** Ali Elbeddini, Thulasika Prabaharan, Sarah Almasalkhi, Cindy Tran

**Affiliations:** 1 Chairman of the Pharmacy, Winchester District Memorial Hospital, 566 Louise Street, Winchester, ON K0C2K0 Canada; 2 Leslie Dan Faculty of Pharmacy, University of Toronto, 144 college st, Toronto, M5S 3M2 Canada

**Keywords:** Frontline health workers, Pharmacist, COVID-19, Pandemic

## Abstract

In the fight against COVID-19, frontline health workers have been vital to keeping the pandemic at bay, but recognition of individual professions’ efforts have been inconsistent at all levels. Pharmacists around the world have continued to provide direct patient care and perform frontline duties for their communities during this pandemic, but are often relegated to the background and overlooked when frontline workers are heralded. Community pharmacists are the most accessible healthcare practitioners, which is further proven during the pandemic as they continued to provide direct patient care despite restrictions imposed by the government due to the pandemic. Due to the inaccessibility of other healthcare practitioners during this time, community pharmacists have reduced the burden on the healthcare system by diverting the influx of patients away from hospitals through triaging and screening patients. Community pharmacists have played various roles in supporting the healthcare system during COVID-19: delivering medications to patients, educating patients on telehealth services, assessing patients for renewal of chronic medications, performing consultations on minor ailments, clarifying misconceptions about COVID-19 treatments, and contributing to COVID-19 screening. Alongside ICU nurses, physicians, and respiratory therapists, hospital pharmacists have been part of the COVID-19 efforts and their roles include management of drug shortages, development of treatment protocols, participation of patient rounds, interpretation of lab results for COVID-19, participant recruitment for clinical trials, exploration of new drugs, medication management advice, and antimicrobial stewardship. Further support from pharmacists will be needed once a vaccine is launched in order to reach population-wide coverage. Amid COVID-19, pharmacists have not stopped working as frontline workers and they should be recognized as such.

## Introduction

Pharmacists should have always been considered frontline workers, but especially amid COVID-19. There is no universal definition of what a frontline worker is, which leads to inconsistency on how to further classify essential workers globally during this pandemic. As a result, governments around the world have varied in how to, or whether to even recognize the work of pharmacists during the pandemic. New Zealand has provided extra remuneration for pharmacists’ contributions amidst COVID-19, while Ontario, Canada’s most populace province, has failed to include pharmacists as part of the list of frontline workers, a list that includes co-workers that many pharmacists share a place of work with. Whether in the community or hospital, pharmacists have been performing frontline roles, yet they are not universally being recognized as such.

## Community pharmacists amid COVID

**Fig. 1:**
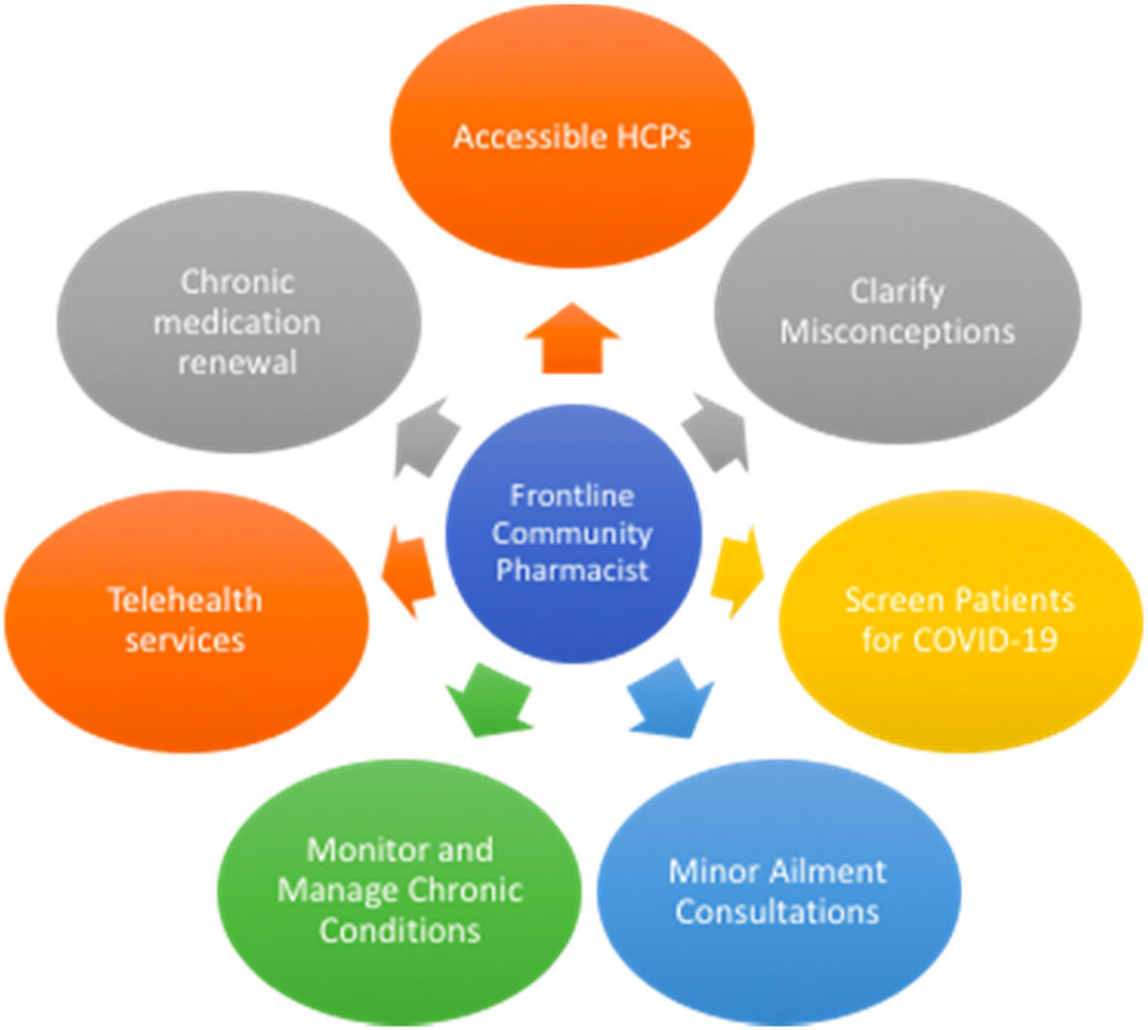
Key Responsibilities of Frontline Community Pharmacists amid COVID-19

Pharmacists have always been the most accessible health care provider; this is especially true in the era of COVID-19. While other professionals have closed their doors to patients, community pharmacies remained open to the public despite stricter lockdown restrictions. As highly trusted healthcare clinicians, community pharmacists play a vital role in closing the gaps that are exacerbated by the additional strain on the system and reduced access to healthcare providers. For low to middle income countries, community pharmacies offer the advantage of medical advice without cost to patients who are unable to afford physician fees [1, 2]. Despite the initial shortage of personal protective equipment, pharmacy staff continued to provide direct patient care. Pharmacies are delivering medications to patients free of charge, educating patients on telehealth services, assessing patients that require renewal of chronic medications, performing consultations on minor ailments, clarifying misconceptions about COVID-19 treatments, and contributing to COVID-19 screening [3]. Fig. 1 summarizes the key responsibilities of frontline community pharmacists. Through their triaging, community pharmacists are maximizing the efficiency of the healthcare system during this time of limited resources.

## Hospital pharmacists amid COVID

**Fig. 2:**
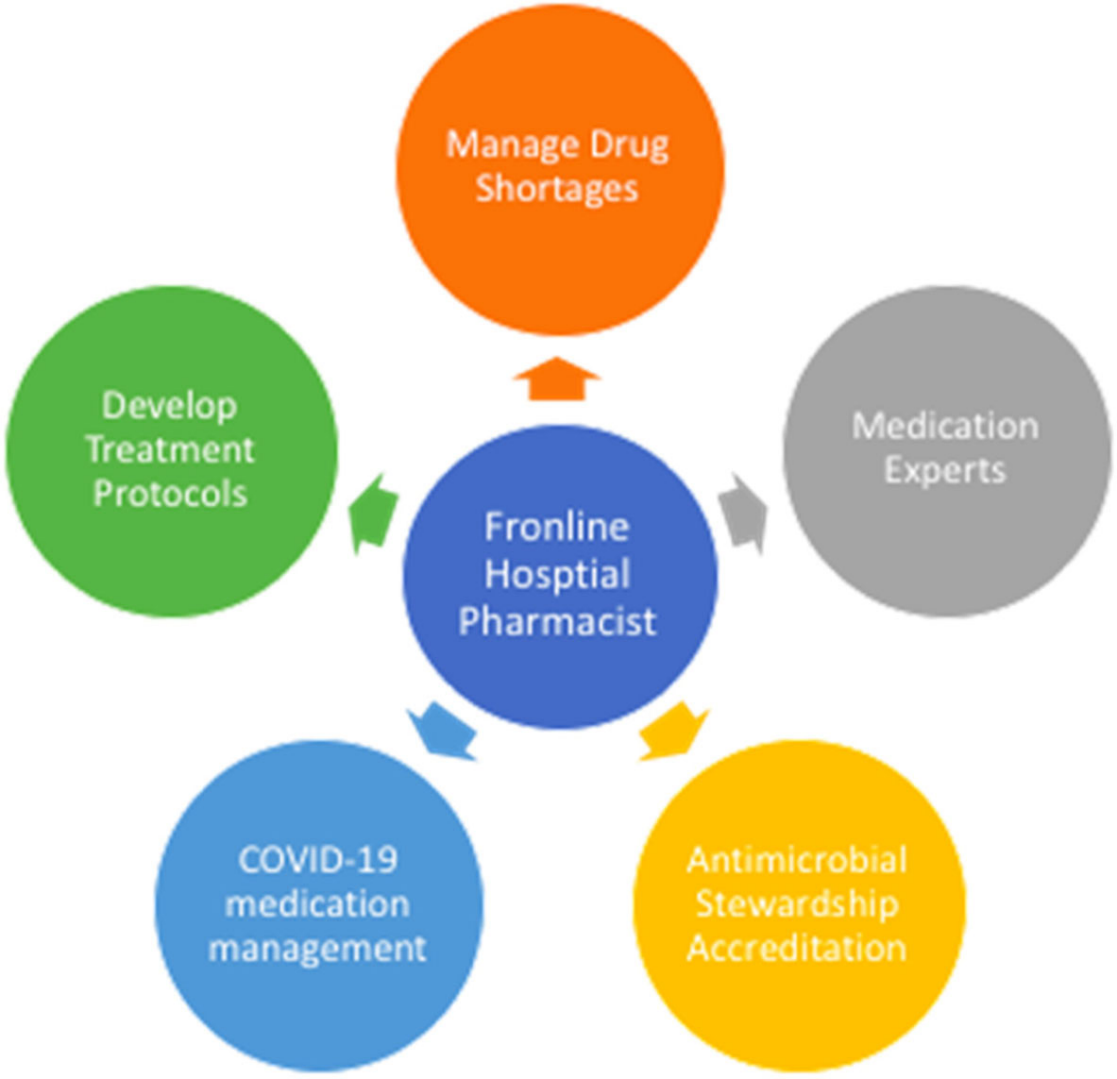
Key Responsibilities of Hospital Pharmacists amid COVID-19

The role of hospital pharmacists during this pandemic is also being overlooked by the public. Alongside ICU nurses, physicians, and respiratory therapists, hospital pharmacists contribute to COVID-19 management protocols by participating in inpatient rounds, ensuring sufficient medication supply to support ICU beds while managing critical drug shortages through the implementation of conservation strategies and sourcing alternatives [4]. Hospital pharmacists participate in antimicrobial stewardship programs; thus are directly involved in planning and responding to pathogen outbreaks, which is of heightened importance during COVID-19. As part of antimicrobial stewardship programs, hospital pharmacists have been involved in developing local treatment protocols that repurpose antivirals and monitoring the use of antibiotics in cases of bacterial co-infections in COVID-19 patients [5]. In addition, pharmacists can help interpret test results for COVID-19, explore new drug therapies or uses, and provide medication management recommendations to their colleagues [6]. While there is no current cure for COVID-19, potential treatment options like hydroxy-chloroquine, methylprednisolone, and remdesivir are being evaluated in clinical trials [6]. Hospital pharmacists can help with the enrolment of infected patients for these types of studies [6]. Fig. 2 describes the key roles of frontline hospital pharmacists. Because of various roles hospital pharmacists play in the effort against COVID-19, they are constantly exposed to the virus. However, they manage to complete their responsibilities similarly to their co-workers without the same recognition as frontline workers.

## The future role of pharmacists amid COVID

Looking forward, pharmacists’ medication expertise should be leveraged in vaccine development and clinical trials. When the vaccine against COVID-19 is available, pharmacists will be considered one of the frontline health workers that should be permitted to give immunizations. Given the past success of community pharmacists with increasing annual seasonal influenza uptake and their accessibility, pharmacists will need to be central in administering COVID-19 vaccines in order to achieve rapid population-wide coverage. Furthermore, screening and testing patients for COVID-19 are both crucial interventions to flatten the curve. Pharmacists in Alberta are screening patients daily and referring them to nearest testing facilities [7]. While American pharmacists may order and administer FDA-approved tests [8]. Increasing the accessibility of testing is imperative if countries wish to escape lockdowns.

## Conclusion

In the fight against COVID-19, our shield is the healthcare system and our soldiers, healthcare professionals, which undoubtedly include pharmacists. Pharmacists have not stopped working because of COVID-19 and in fact, have stepped up to take on more responsibilities. Their efforts should not be forgotten when frontline workers are lauded once this global pandemic ends, but without question, should not be overlooked in the present, when their frontline efforts are still needed to fight COVID-19. Pharmacists are frontline workers; they should be addressed as such and given the recognition they deserve.

## Data Availability

Data sharing does not apply to this article as no datasets were generated or analyzed during the current study.
